# Real-World Survival Outcomes Following Chemoradiotherapy with or Without Durvalumab in PD-L1-Defined Subgroups of Stage III Unresectable NSCLC

**DOI:** 10.3390/curroncol33060351

**Published:** 2026-06-10

**Authors:** Janne Selke, Yvonne Dzierma, Katrin Manda, Guido Hildebrandt, Bernd Frerker, Paul Kalata, Christin Ackermann, Felix Bock

**Affiliations:** 1 Department of Radiotherapy and Radiation Oncology, Rostock University Medical Centre, 18059 Rostock, Germany; janne.selke@uni-rostock.de (J.S.); yvonne.dzierma@med.uni-rostock.de (Y.D.); katrin.manda@med.uni-rostock.de (K.M.); guido.hildebrandt@med.uni-rostock.de (G.H.); bernd.frerker@med.uni-rostock.de (B.F.); paul.kalata@med.uni-rostock.de (P.K.); 2 Institute of Hematopathology Hamburg HpH, 22547 Hamburg, Germany; ackermann@hp-hamburg.de

**Keywords:** locally advanced non-small cell lung cancer, stage III, PD-L1, durvalumab, chemoradiotherapy, real-world evidence

## Abstract

Durvalumab consolidation after definitive chemoradiotherapy (CRT) is the standard of care for unresectable stage III non-small cell lung cancer (NSCLC) but real-world data comparing PD-L1-defined subgroups are limited. This retrospective single-centre study analyzed overall survival (OS) and progression-free survival (PFS) in 142 patients with inoperable stage III NSCLC with definitive CRT between 2015 and 2022. All tumours were assessed for PD-L1 expression, including retrospective testing, where required. Patients were assigned into three cohorts: PD-L1-positive with (PD-L1+mD; *n* = 57) and without (PD-L1+oD; *n* = 44) durvalumab maintenance and PD-L1-negative without durvalumab (PD-L1−oD; *n* = 41). Mean follow-up was 44.0 months. Median OS was 27.3, 15.1 and 23.4 months for PD-L1+mD, PD-L1+oD, and PD-L1−oD, respectively. Median PFS was 18.4, 10.5 and 13.4 months for PD-L1+mD, PD-L1+oD, and PD-L1−oD, respectively. OS and PFS were significantly improved with durvalumab in PD-L1-positive patients (*p* = 0.043 and *p* = 0.027). PD-L1-negative patients showed no significant OS or PFS differences versus PD-L1+oD. Immunotherapy-related pneumonitis ≥grade 1 was documented in 15.7% patients. In real-world practice, durvalumab improves OS and PFS in PD-L1-positive unresectable stage III NSCLC and its omission appears particularly unfavourable.

## Simple Summary

Durvalumab after chemoradiotherapy is standard treatment for unresectable stage III non-small cell lung cancer, but direct real-world comparison across PD-L1-defined subgroups remain limited. In this study, we analyzed overall and progression-free survival in routine clinical practice. Patients with PD-L1-positive tumours who received durvalumab had significantly longer survival than those treated without it. In contrast, PD-L1-positive patients who did not receive durvalumab showed the shortest survival outcomes. These results support the effectiveness of durvalumab in routine care and emphasize the importance of PD-L1 testing and access to consolidation immunotherapy in daily practice.

## 1. Introduction

Lung cancer, with approximately 2.5 million new cases annually, remains the leading cause of cancer-related mortality worldwide [1]. In Germany alone, about 58.400 individuals were diagnosed with lung cancer in 2023 [2]. Lung cancer currently represents the most common cause of cancer death in men and the second most common in women in Germany [2]. Non-small cell lung cancer (NSCLC) accounts for around 80% of all lung cancer cases and is associated not only with substantial individual morbidity and mortality, but also with considerable social and economic burdens across Europe [3].

Owing to the lack of effective early detection strategies and often subtle initial symptoms, lung carcinomas are frequently diagnosed at advanced stages, so that a relevant proportion of patients is already oncologically unresectable at first presentation [4]. Furthermore, patients often present with comorbidities associated with reduced respiratory function, rendering them functionally inoperable. For this substantial patient collective of unresectable, locally advanced stage III NSCLC, concurrent chemoradiotherapy (CRT) constitutes an integral component of the standard of care [5,6,7].

In recent years, immunotherapy has emerged as the fourth pillar of multimodal cancer therapy, complementing surgery, chemotherapy and radiotherapy. For patients with unresectable, locally advanced stage III NSCLC, CRT followed by consolidation immunotherapy has substantially improved outcomes. The phase III PACIFIC trial established consolidation therapy with the anti-PD-L1 antibody durvalumab after definitive CRT as a new standard of care, demonstrating significant improvements in progression-free survival (PFS) and overall survival (OS) compared with placebo [8,9]. The observational PACIFIC-R study subsequently confirmed the efficacy and feasibility of durvalumab in a real-world setting across multiple countries [10]. Additionally, several contemporary real-world cohorts from Europe, Canada and Australia have further confirmed the effectiveness and feasibility of durvalumab consolidation after definitive CRT in clinical routine and have shown improved outcomes with increasing PD-L1 expression, thereby supporting the clinical relevance of PD-L1 in this setting [11,12,13,14].

The clinical benefit of durvalumab is closely linked to tumour immune biology, in particular the programmed death-1 (PD-1)/programmed death-ligand 1 (PD-L1) axis. In Europe, and specifically in Germany, approval and reimbursement for durvalumab consolidation after CRT in stage III NSCLC are restricted to tumours exhibiting pretherapeutic PD-L1 expression according to the European Medicines Agency (EMA) label and national guideline recommendations [5].

While randomized clinical trial data from PACIFIC have clearly defined durvalumab consolidation as the standard of care, it remains crucial to understand its effectiveness and safety under routine clinical conditions, particularly in relation to the PD-L1 status. Real-world cohorts frequently differ from trial populations with respect to comorbidities, performance status and treatment adherence. Moreover, direct comparison between PD-L1-positive patients who do and do not receive durvalumab consolidation and PD-L1-negative patients treated with definitive CRT alone remain limited, especially in monocentric cohorts with complete PD-L1 characterization of all patients.

Given these considerations, additional real-world data directly comparing PD-L1-defined patient groups may help to further clarify the impact of PD-L1 status and durvalumab use on survival outcomes in routine clinical practice and complement evidence derived from existing trials.

Therefore, the present retrospective, single-centre study aimed to evaluate OS and PFS in inoperable stage III NSCLC patients treated with definitive chemoradiotherapy in German routine clinical practice between 2015 and 2022. All patients were characterized for PD-L1 status, including retrospective testing where required. We specifically compared three cohorts according to PD-L1 status and use of durvalumab consolidation: PD-L1-positive patients with durvalumab maintenance, PD-L1-positive patients without durvalumab, and PD-L1-negative patients without durvalumab.

## 2. Materials and Methods

### 2.1. Patients

Clinical data of 142 patients with unresectable stage III non-small cell lung cancer (NSCLC) treated with definitive, concomitant chemoradiotherapy (CRT) between 2015 and 2022 at the Department of Radiotherapy and Radiation Oncology, University Medical Centre Rostock, were retrospectively analyzed. The majority of cases (109/142) were discussed in a multidisciplinary tumour board consisting of at least thoracic surgeons, medical oncologists, pulmonologists, radiologists, radiation oncologists and pathologists. Unresectability was determined by multidisciplinary consensus based on tumour extent, nodal involvement, expected surgical morbidity and patient-related factors, taking into account thoracic surgical evaluation. Patients who underwent surgical resection or were treated within a perioperative treatment strategy were not included in this analysis.

Inclusion criteria were histologically confirmed primary stage III NSCLC at the initial staging between 2015 and 2022, an indication for definitive CRT and availability of tumour tissue for PD-L1 assessment. For all patients with initially unknown PD-L1 status (*n* = 12), PD-L1 expression was determined retrospectively by re-examining primarily taken tumour tissue obtained at first diagnosis. Among the 101 PD-L1-positive tumours, antibody clones could be identified in 40 cases. The clinically validated clones 22C3 (*n* = 22), E1L3N (*n* = 16) and 73–10 (*n* = 2) were used. For the remaining 61 cases (60.4%), the originally used antibody clone could not reliably reconstruct from the available documentation. Regardless of the assay used, PD-L1 positivity was consistently defined as ≥1% tumour cell staining. In addition to binary classification, PD-L1 expression levels were further categorized as shown in Table 1. The number of patients with very high PD-L1 expression was limited and therefore not analyzed separately.

Baseline demographic and clinical characteristics were extracted from electronic medical records, including age, sex, Karnofsky performance status and smoking history. Tumour-related factors comprised histological subtype, UICC stage according to the 8th edition of the TNM classification and PD-L1 expression status.

The patients were assigned into three cohorts according to PD-L1 status and receipt of durvalumab maintenance: PD-L1-positive with durvalumab (PD-L1+mD), PD-L1-positive without durvalumab (PD-L1+oD) and PD-L1-negative without durvalumab (PD-L1−oD).

### 2.2. Treatment and Toxicity Assessment

Radiotherapy was delivered using 6 MV photons with either intensity-modulated radiotherapy (IMRT) or volumetric modulated arc therapy (VMAT) with image guidance according to institutional standards. Gross tumour volume (GTV), clinical target volume (CTV) and planning target volume (PTV) were contoured on planning CT scans with incorporation of pretherapeutic FDG-PET/CT imaging. The prescribed total radiation dose was typically 60–66 Gy (average (AVG)) in 2.0 Gy (AVG) fractions to the PTV.

**Figure 1:**
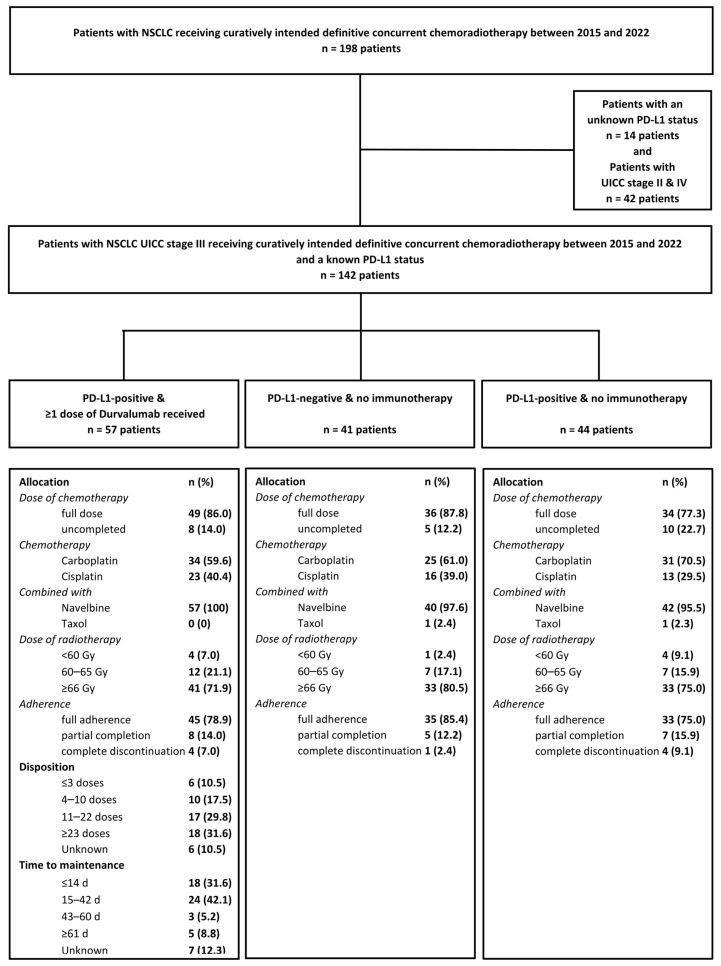
Flowchart with treatment characteristics of all three groups (*n* = 142).

Concurrent chemotherapy regimens were platinum-based (Figure 1) and administered according to current guidelines. Complete concurrent chemotherapy was defined as the administration of at least 80% of two planned cycles of platinum-based chemotherapy during radiotherapy.

Durvalumab maintenance was restricted to PD-L1-positive patients (≥1%) who completed CRT after regulatory approval and reimbursement of durvalumab in Germany, provided no disease progression was determined after the completion of CRT. In our institutional workflow, since weekly cone beam computed tomography (CBCT) imaging is performed during radiotherapy, the last CBCT was assessed to determine tumour response. For 42 of 50 (84.0%) documented patients with at least stable disease, maintenance therapy was initiated within 42 days from the last radiotherapy fraction. In 8 cases, durvalumab was started more than 42 days after the last fraction and in 7 cases, documentation regarding maintenance therapy period remained incomplete. The patients treated before the regulatory approval of durvalumab and those with negative PD-L1 status received CRT alone without consolidation immunotherapy. The patients in the PD-L1+mD group were predominantly treated from 2018 onwards, whereas those in the PD-L1+oD group were mainly treated before this time point. Radiotherapy techniques (IMRT vs. VMAT) and the use of FDG-PET/CT for staging were reviewed and described.

### 2.3. Study Design and Ethics

This study is a retrospective, single-centre analysis of prospectively documented clinical routine data. Treatment decisions, including the indication for CRT and durvalumab maintenance were made independently of this analysis and in accordance with contemporary guidelines and the treating physicians’ judgement.

The study was conducted in accordance with the Declaration of Helsinki and approved by the Institutional Review Board of the University Medical Centre Rostock (A2024-0010).

### 2.4. Endpoints and Follow-Up

The primary endpoints were overall survival (OS) and progression-free survival (PFS). OS was defined as the time from histological confirmation to death from any cause or last follow-up. PFS was defined as the time from histological confirmation to the first documented event of locoregional progression, distant metastasis or death, whichever occurred first. Patients without an event were censored at the date of last follow-up. The mean follow-up was 44.0 months (range 1.4–90.8 months).

Toxicity data were retrospectively collected from medical reports during concurrent chemoradiotherapy, during durvalumab maintenance (if applicable) and during follow-up visits after completion of treatment. Missing data, particularly for patients receiving durvalumab were obtained from external treating physicians. Adverse events were classified according to CTCAE version 5.0.

### 2.5. Statistical Analysis

Survival curves were estimated using the Kaplan–Meier method and compared between groups using the log rank test. Hazard ratios (HRs) and 95% confidence intervals (CI) were calculated using Cox proportional hazards model. Categorical variables were compared using the Pearson’s chi-square test, Fisher’s exact test or Fisher–Freeman–Halton exact test. Continuous variables were summarized as median and range and compared using the Kruskal–Wallis test. A *p*-value of <0.05 was considered statistically significant. The median follow-up was calculated using the reverse Kaplan–Meier method. Statistical analyses were performed using IBM SPSS Statistics, v29.0.1.1.

## 3. Results

### 3.1. Patient Cohort and Baseline Characteristics

A total of 142 patients with unresectable stage III NSCLC treated with definitive CRT between 2015 and 2022 were included. Of these, 57 patients were PD-L1-positive and received durvalumab maintenance (PD-L1+mD), 44 were PD-L1-positive without durvalumab (PD-L1+oD) and 41 were PD-L1-negative without durvalumab (PD-L1−oD). Baseline patient and tumour characteristics are summarized in Table 1. The three cohorts were generally well balanced regarding age, sex, Karnofsky performance status, smoking status, histology, UICC stage, use of pretherapeutic FDG-PET/CT and days between histological confirmation to start of CRT. Only radiotherapy technique differed significantly between groups (*p* < 0.001).

### 3.2. Treatment Characteristics

Most patients received a total radiation dose of 66 Gy (AVG) with concurrent platinum-based chemotherapy. Durvalumab maintenance was administered to all PD-L1-positive patients treated after regulatory approval. In 51 of 57 (89.5%) patients, the exact number of cycles and the occurrence of adverse events were documented. The reasons for discontinuation of durvalumab maintenance included disease progression (*n* = 15), pneumonia (*n* = 4), deterioration in performance status (*n* = 3), death (*n* = 2), colitis (*n* = 1), thrombocytopenia (*n* = 1), heart failure (*n* = 1), myopathy (*n* = 1) and pulmonary embolism (*n* = 1). The median number of durvalumab cycles was 16 (range 1–33). Treatment characteristics are summarized in Figure 1.

### 3.3. Overall Survival

**Figure 2:**
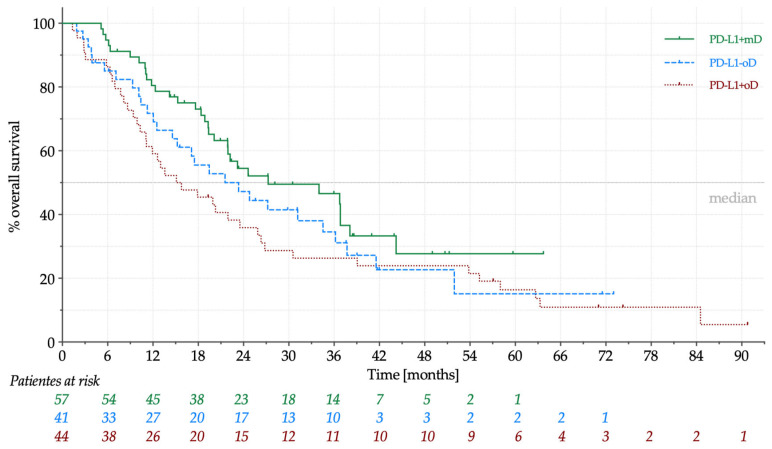
Kaplan–Meier plot of overall survival (OS) in 142 patients with inoperable NSCLC treated with definitive CRT. Green: PD-L1-positive patients with durvalumab (PD-L1+mD) (*n* = 57), blue: PD-L1-negative patients without durvalumab (PD-L1−oD) (*n* = 41), red: PD-L1-positive patients without durvalumab (PD-L1+oD) (*n* = 44).

Median overall survival was 27.3 months (95% CI: 12.2–42.4) in the PD-L1+mD group, 15.1 months (95% CI: 7.2–23.0) in the PD-L1+oD group and 23.4 months (95% CI: 12.8–33.9) in the PD-L1−oD group (Figure 2). PD-L1-positive patients receiving durvalumab maintenance had significantly improved OS compared with PD-L1-positive patients without durvalumab (*p* = 0.043) resulting in a hazard ratio of 0.61 (95% CI: 0.39–0.99). OS did not differ significantly between PD-L1-negative patients and either PD-L1-positive patients without durvalumab (*p* = 0.476) or PD-L1-positive patients with durvalumab (*p* = 0.217).

Multivariable Cox regression confirmed durvalumab consolidation (HR 0.58, 95% CI 0.36–0.96, *p* = 0.035) and a KPS ≥80 (HR 0.60, 95% CI 0.37–0.98, *p* = 0.043) as independent predictors of improved overall survival. Male sex (HR 1.7, 95% CI 1.03–2.9, *p* = 0.039) and squamous histology (HR 1.65, 95% CI 1.04–2.62, *p* = 0.034) were independently associated with poorer survival outcomes. The UICC stage, pretherapeutic FDG-PET/CT, radiotherapy technique and age were not significantly associated with overall survival.

### 3.4. Progression-Free Survival

**Figure 3:**
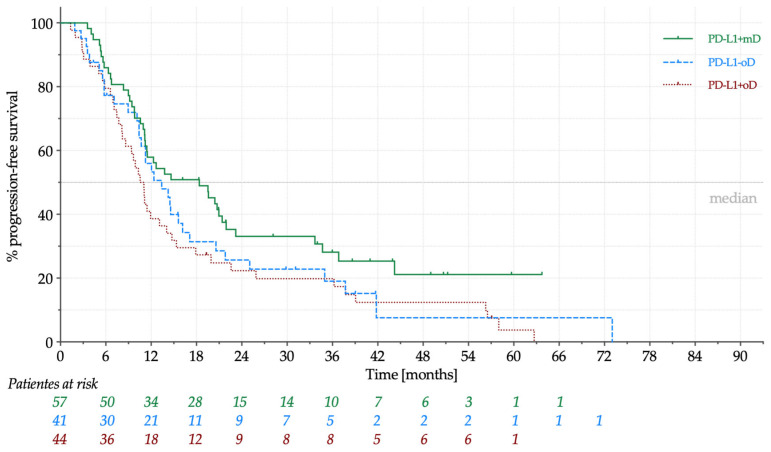
Kaplan–Meier plot of progression-free survival (PFS) in 142 patients with inoperable NSCLC treated with definitive CRT. Green: PD-L1-positive patients with durvalumab (PD-L1+mD) (*n* = 57), blue: PD-L1-negative patients without durvalumab (PD-L1−oD) (*n* = 41), red: PD-L1-positive patients without durvalumab (PD-L1+oD) (*n* = 44).

Median PFS was 18.4 months (95% CI: 10.1–26.6), 10.5 months (95% CI: 8.9–12.1) and 13.4 months (95% CI: 9.6–17.2) for the PD-L1+mD, PD-L1+oD and PD-L1−oD groups, respectively (Figure 3). PD-L1-positive patients receiving durvalumab maintenance had significantly improved PFS compared with PD-L1-positive patients without durvalumab (*p* = 0.027) resulting in a hazard ratio of 0.62 (95% CI: 0.40–0.95). PFS did not differ significantly between PD-L1-negative patients and either PD-L1-positive patients without durvalumab (*p* = 0.421) or PD-L1-positive patients with durvalumab (*p* = 0.176). Multivariable Cox regression confirmed durvalumab consolidation (HR 0.61, 95% CI 0.39–0.94, *p* = 0.027) and a KPS ≥ 80 (HR 0.56, 95% CI 0.36–0.86, *p* = 0.013) as independent predictors of improved PFS. No significant associations were observed for sex, histology, disease stage, pretherapeutic FDG-PET/CT, radiotherapy technique, or age.

Local progression occurred in 7/57 (12.3%), 2/44 (4.5%) and 4/41 (9.8%) patients in the PD-L1+mD, PD-L1+oD and PD-L1–oD groups, respectively, while distant progression was observed in 22/57 (38.6%), 26/44 (36.4%) and 16/41 (39.0%) patients in these groups.

### 3.5. Toxicity

All adverse events between the three groups are summarized in Table 2. Rates of esophagitis, hematologic toxicity and other adverse events did not differ significantly between patient groups. Complete documentation regarding the occurrence of adverse events during maintenance therapy with durvalumab was available for 51 of the 57 treated patients. Documented immunotherapy-related pneumonitis ≥grade 1 occurred in 8/51 (15.7%) patients treated with durvalumab. Treatment discontinuation of durvalumab maintenance due to adverse events occurred in 12 of 51 (23.5%) patients.

## 4. Discussion

In our retrospective single-centre real-world cohort of unresectable stage III NSCLC patients with definitive CRT, we observed a clinically relevant and statistically significant improvement in both overall survival and progression-free survival for PD-L1-positive patients receiving durvalumab maintenance compared with PD-L1-positive patients treated with CRT alone. These findings are in line with the pivotal PACIFIC trial, which demonstrated OS and PFS benefits with durvalumab consolidation after definitive CRT [8,9] and with the PACIFIC-R observational study, which confirmed feasibility and effectiveness of durvalumab in routine clinical practice across multiple countries [10]. Our data extend these observations by providing a direct comparison of three clinically relevant subgroups, PD-L1-positive with durvalumab, PD-L1-positive without durvalumab and PD-L1-negative without durvalumab, within a homogeneous treatment setting at our German tertiary centre.

Notably, with a median overall survival of 27.3 months in the PD-L1+mD group and 15.1 months in the PD-L1+oD group, our results are clearly inferior to the OS rates of 47.5 and 29.1 months reported in the PACIFIC trial [9]. Real-world data from the United States likewise demonstrated higher median OS values of 50.2 and 11.6 months for PD-L1-positive patients treated with and without durvalumab [15] and a cohort from Quebec reported median OS of 52.8 and 19 months, respectively [16]. Similarly, recent real-world cohorts from France and Australia reported 3-year OS rates ranging from approximately 55% to 61.1% after durvalumab consolidation [11,13]. In the Canadian study, median OS improved from 21.3 months after CRT alone to 44.6 months after CRT followed by durvalumab, with more favourable outcomes observed in patients with higher PD-L1 expression [14].

While OS in our PD-L1+oD group is in a similar range to the non-durvalumab cohorts in these real-world studies, the survival of our PD-L1+mD patients is considerably shorter. This likely reflects a less selected, more comorbid population and differences in staging and post-progression therapy, rather than a fundamentally reduced efficacy of durvalumab in our setting. While PACIFIC predominantly enrolled patients with ECOG 0–1, our cohort may include a higher proportion of patients with impaired performance (median Karnofsky performance status 80%), which is only partially captured in routine documentation and may not be fully accounted for in our analyses. Differences in histologic subtypes may also contribute. The PACIFIC trial included a higher proportion of non-squamous carcinomas (54.3%) compared with our PD-L1-positive cohort (36.8%) [17]. Furthermore, stage distribution differed. The PACIFIC trial predominantly enrolled patients with stage IIIA (52.9%) and IIIB (44.7%), whereas our real-world PD-L1-positive cohort included a higher proportion of stage IIIB (61.4%) and IIIC (17.5%) tumours [17]. Furthermore, durvalumab discontinuation due to adverse events occurred in 23.5% of cases in our study, compared with only 15.4% in the PACIFIC trial. Finally, EGFR mutation testing was not routinely performed in our cohort, which may further limit direct comparability with PACIFIC and other contemporary series.

Although PD-L1-negative patients treated with CRT alone demonstrated numerically longer OS than PD-L1-positive patients without durvalumab, these differences were not statistically significant. Given the limited sample size of the individual subgroups, no firm conclusion regarding a potential prognostic effect of PD-L1 negativity can be drawn from our data. Nevertheless, the observed outcome pattern is consistent with previous reports suggesting that PD-L1 expression may influence prognosis after chemoradiotherapy, although published evidence remains heterogeneous [18,19]. Biological differences between PD-L1-positive and -negative tumours may contribute. Biologically, PD-L1 positivity may reflect adaptive immune resistance, in which tumour cells upregulate PD-L1 in response to interferon-γ released by activated T cells. In the absence of checkpoint blockade, this mechanism may facilitate immune escape and contribute to inferior outcomes [20]. In addition, PD-L1-positive tumours may be associated with a more immunosuppressive tumour microenvironment, including exhausted T cells and regulatory immune cell populations, and may also reflect more aggressive intrinsic tumour biology driven by oncogenic signalling pathways [21,22]. These mechanisms could further explain why PD-L1-positive patients without durvalumab showed numerically poorer outcomes than PD-L1-negative patients in our cohort. However, differences did not reach statistical significance in our analysis. Moreover additional evidence regarding the independent prognostic role of PD-L1 after CRT remains heterogeneous, therefore these findings must be interpreted with caution [23,24].

Nonetheless, our findings underline that PD-L1-positive patients appear to derive substantial benefit from durvalumab in routine care and that omission of consolidation immunotherapy in this subgroup is associated with inferior overall survival outcomes. Noteworthy, restrictions regarding durvalumab maintenance to PD-L1-positive patients is worth discussing, as trials could also proof benefit for PD-L1-negative patients in a real-world setting [14,25].

We also observed statistically significant (*p* = 0.027) differences in median progression-free survival between PD-L1-positive patients with and without durvalumab (18.4 and 10.5 months, respectively). Median PFS in PD-L1-negative patients was 13.4 months. French real-world cohorts reported median PFS values between 18.5 and 22.6 months for PD-L1-positive patients with durvalumab, while the Australian cohort reported a median PFS of 22.4 months [11,13]. Furthermore, French PACIFIC-R data demonstrated longer PFS in PD-L1-positive compared with PD-L1-negative tumours, with median PFS of 25.3 months versus 13.0 months, further supporting the clinical relevance of PD-L1 expression in this treatment setting [11]. Thus, our findings align with the PACIFIC trial and other contemporary real-world analyses, showing statistically significant PFS differences between PD-L1-positive patients with and without durvalumab [9,15,16,17,26,27,28]. Consistent with our overall survival data, PFS in PD-L1-negative patients treated with CRT alone compared to PD-L1-positive patients without durvalumab did not differ significantly.

While PFS assessment in real-world settings may be affected by variability in imaging intervals and response evaluation [29,30,31], the consistency of our findings with randomized data supports that OS and PFS are crucial endpoints for evaluating the benefit of durvalumab in this setting.

From a health care delivery perspective, our data suggest that durvalumab has been successfully implemented in routine clinical practice. Following regulatory approval and reimbursement in Germany all eligible PD-L1-positive patients in our cohort received durvalumab consolidation. This high uptake indicates adherence to guideline recommendations and demonstrates the feasibility of integrating durvalumab into everyday workflows. At the same time, the large group of PD-L1-positive patients without durvalumab from pre-approval era provides an internal comparator that highlights the magnitude of the survival benefit associated with durvalumab in real-world practice.

Regarding safety, the incidence of immunotherapy-related pneumonitis ≥grade 1 in our cohort was within the range reported in PACIFIC-R and other analyses [11,13,16,17,26]. The rates of esophagitis, hematologic toxicity and other events did not differ between patients with and without durvalumab. These findings support the overall tolerability of durvalumab consolidation following definitive CRT in an unselected clinical population.

As patients were treated over an extended period, temporal aspects should be considered. While the use of pretherapeutic FDG-PET/CT was comparable across all three groups, radiotherapy techniques differed between cohorts, with IMRT being more frequently applied in the PD-L1+oD group and VMAT predominantly used in the PD-L1+mD group, reflecting the evolution of clinical practice over time. However, previous studies have not demonstrated a significant impact of radiotherapy technique on survival outcomes [32,33]. Consistent with these findings, radiotherapy technique was not independently associated with either OS or PFS in our multivariable Cox regression analyses.

Our study has several strengths and limitations that should be considered when interpreting the results. Strengths include a well-defined, homogeneous cohort of multidisciplinary assessed unresectable stage III NSCLC patients treated with curatively intended contemporary radiation techniques and platinum-based CRT at a single German tertiary centre, as well as complete PD-L1 characterization of all patients, including retrospective testing of primary specimen where necessary. This allows a clear stratification by PD-L1 status and durvalumab use.

On the other hand, the retrospective, single-centre design inherently carries risks of unmeasured confounders. Although baseline characteristics were generally balanced between cohorts’ subtle differences in comorbidities and missing data may have influenced outcomes. Toxicity reporting may also be limited. While efforts were made to collect adverse events from institutional and external records, particularly during durvalumab maintenance, underreporting cannot be excluded. Additionally, a potential for temporal bias needs to be mentioned. In particular, PD-L1-positive patients who did not receive durvalumab were treated before the implementation of the PACIFIC regimen in routine clinical practice, whereas the durvalumab-treated patients were treated thereafter. Consequently, differences in staging procedures, supportive care and treatment delivery associated with evolving clinical practice cannot be completely excluded. PD-L1 assessment in our cohort was partly retrospective (*n* = 12) and based on archived tissue samples. This may introduce variability due to differences in antibody clones, testing platforms, sample age and tissue origin. Furthermore, because of the study period and the retrospective collection of data, the originally used PD-L1 assay could no longer be reliably reconstructed for all samples. Particularly, older specimens may underestimate PD-L1 expression, which could have influenced group allocation. The sample size, while comparable to other monocentric series, limits statistical power. OS and PFS were calculated from the date of histological confirmation rather than from later treatment-related time points used in discussed studies. Although this may limit direct comparability with external cohorts, the interval between histological confirmation and treatment initiation did not differ significantly between our study groups and is therefore unlikely to affect the internal comparative validity of our analyses. Finally, follow-up duration may still be insufficient to fully capture very long-term results.

In summary, our real-world data confirm that survival benefit for durvalumab consolidation observed in the PACIFIC trial translates into routine clinical practice for PD-L1-positive unresectable stage III NSCLC. PD-L1-positive patients who do not receive durvalumab appear to have the shortest survival, whereas outcomes for PD-L1-negative patients treated with CRT alone are numerically better without statistical significance. Larger, preferably multicentre real-world studies with detailed adjustments for confounders and longer follow-up, as well as studies exploring the potential of liquid biopsy to detect dynamic changes in PD-L1 expression on circulating tumour cells [34,35], are needed to further refine patient selection and to better understand the interaction between CRT and PD-L1 status.

## 5. Conclusions

In this retrospective single-centre real-world cohort of patients with unresectable stage III NSCLC treated with definitive chemoradiotherapy, PD-L1-positive patients who received durvalumab maintenance showed a clinically relevant and statistically significant improvement in overall survival and progression-free survival compared with PD-L1-positive patients without consolidation immunotherapy. Outcomes of PD-L1-negative patients treated with CRT alone were comparable to those of PD-L1-positive patients who did not receive durvalumab, suggesting that omission of durvalumab may be particularly detrimental in PD-L1-positive disease. After regulatory approval, durvalumab was successfully implemented in routine clinical practice for eligible PD-L1-positive patients. Overall, our data support the use of durvalumab maintenance as the standard of care for PD-L1-positive stage III NSCLC in routine clinical practice and underscore the central role of PD-L1 testing.

## Figures and Tables

**Table 1:** Clinical baseline characteristics of patients with stage III unresectable non-small cell lung cancer (NSCLC) and tumour-related variables. Demographic data, disease characteristics and tumour features are summarized at baseline for the study cohort. Values are reported as *n* (%) or median (range), as appropriate. PD-L1 positivity was defined as ≥1% throughout the study. The additional PD-L1 subcategories (<25%, 25–49%, and ≥50%) are presented for descriptive purposes and were not used for outcome analyses.

		PD-L1+mD, *n* = 57	PD-L1+oD, *n* = 44	PD-L1−oD, *n* = 41	*p*-Value
Sex	Male, *n* (%)	39 (68.4)	31 (70.5)	33 (80.5)	0.390
	Female, *n* (%)	18 (31.6)	13 (29.5)	8 (19.5)	
Age (years)	Median (range)	64.58 (45.01–78.09)	63.57 (46.82–77.49)	63.54 (44.75–80.84)	0.699
Karnofsky performance status	Median (range)	80 (70–100)	80 (70–100)	80 (70–100)	0.086
Smoking status, *n* (%)	Never	4 (7.0)	2 (4.5)	3 (7.3)	0.819
	Former	22 (38.6)	14 (31.8)	17 (41.5)	
	Active	31 (54.4)	28 (63.6)	21 (51.2)	
Histology, *n* (%)	Squamous cell carcinoma	36 (63.2)	26 (59.1)	23 (56.1)	0.775
	Non-squamous cell carcinoma	21 (36.8)	18 (40.9)	18 (43.9)	
PD-L1 status, *n* (%)	PD-L1 <25%	29 (50.9)	28 (63.6)	-	0.127
	PD-L1 >25–49%	14 (24.6)	4 (9.1)	-	
	PD-L1 ≥50%	14 (24.6)	12 (27.3)	-	
UICC stage 8th edition, *n* (%)	IIIA	12 (21.1)	17 (38.6)	14 (34.1)	0.380
	IIIB	35 (61.4)	21 (47.7)	22 (53.7)	
	IIIC	10 (17.5)	6 (13.6)	5 (12.2)	
Pretherapeutic FDG-PET/CT	Received, *n* (%)	56 (98.2)	40 (90.9)	39 (95.1)	0.240
	Not received, *n* (%)	1 (1.8)	4 (9.1)	2 (4.9)	
Days from histological confirmation to CRT	Median (range)	51 (6–167)	40.5 (8–151)	55 (10–152)	0.234
Radiation technique	VMAT, *n* (%)	53 (93.0)	13 (29.5)	26 (63.4)	<0.001
	IMRT, *n* (%)	4 (7.0)	31 (70.5)	15 (36.6)	

CRT: chemoradiotherapy; PD-L1: programmed death-ligand 1; mD: with durvalumab; oD: without durvalumab; UICC: Union for International Cancer Control.

**Table 2:** Summary of adverse events among three NSCLC patient groups: PD-L1-positive patients treated with durvalumab (PD-L1+mD), PD-L1-positive patients without durvalumab (PD-L1+oD) and PD-L1-negative patients without durvalumab. Adverse events are reported as frequencies and grades according to the applied toxicity classification.

	CTCAE Grade	PD-L1+mD, *n* = 57	PD-L1+oD, *n* = 44	PD-L1−oD, *n* = 41
Anemia, *n* (%)	1	2 (3.5)	1 (2.3)	1 (2.4)
	2	25 (43.9)	24 (54.5)	17 (41.5)
	3	30 (52.6)	19 (43.2)	23 (56.1)
Leukocytopenia, *n* (%)	1	19 (33.3)	9 (20.5)	12 (29.3)
	2	19 (33.3)	15 (34.1)	13 (31.7)
	3	7 (12.3)	10 (22.7)	6 (14.6)
Thrombocytopenia, *n* (%)	1	7 (12.3)	7 (15.9)	2 (4.9)
	2	5 (8.8)	6 (13.6)	2 (4.9)
	3	3 (5.3)	2 (4.5)	0 (0.0)
	4	1 (1.8)	0 (0.0)	0 (0.0)
Esophagitis, *n* (%)	described	0 (0.0)	5 (11.4)	1 (2.4)
Dysphagia, *n* (%)	1	20 (35.1)	15 (34.1)	13 (31.7)
	2	8 (14.0)	2 (4.5)	1 (2.4)
	3	0 (0.0)	0 (0.0)	1 (2.4)
	4	0 (0.0)	0 (0.0)	1 (2.4)
	unknown	0 (0.0)	2 (4.5)	1 (2.4)
Fatigue, *n* (%)	1	10 (17.5)	7 (15.9)	11 (26.8)
	2	4 (7.0)	3 (6.8)	4 (9.8)
	unknown	0 (0.0)	2 (4.5)	2 (4.9)
Radiotherapy-related pneumonitis, *n* (%)	described	2 (3.5)	1 (2.3)	0 (0.0)
Immunotherapy-related pneumonitis, *n* (%)	described	8/51 (15.7)	-	-

PD-L1: programmed death-ligand 1; mD: with durvalumab; oD: without durvalumab; UICC: Union for International Cancer Control; CTCAE: Common Terminology Criteria for Adverse Events, version 5.

## Data Availability

The datasets generated and analyzed during the current study are available from the corresponding author on reasonable request.

## Abbreviations

The following abbreviations are used in this manuscript:

AVG	average
CI	confidence interval
CRT	chemoradiotherapy
CTCAE	Common Terminology Criteria for Adverse Events
CTV	clinical target volume
GTV	Gross tumour volume
HR	hazard ratio
IMRT	intensity-modulated radiotherapy
KPS	Karnofsky performance status
mD	with Durvalumab
NSCLC	non-small cell lung cancer
oD	without durvalumab
OS	overall survival
PD-L1	programmed death-ligand 1
PFS	progression-free survival
PTV	planning target volume
UICC	Union for International Cancer Control
VMAT	volumetric modulated arc therapy
